# Evaluation of fully automated chemiluminescent enzyme immunoassays for hepatitis B core-related antigen components, phosphorylated and non-phosphorylated hepatitis B core antigens: clinical significance and dynamics during hepatitis B e antigen seroconversion

**DOI:** 10.1128/jcm.00385-25

**Published:** 2025-08-19

**Authors:** Takanori Suzuki, Chiharu Ohue, Osamu Arai, Yuka Inose, Katsuya Nagaoka, Shintaro Ogawa, Takako Inoue, Kentaro Matsuura, Katsumi Aoyagi, Shintaro Yagi, Yasuhito Tanaka

**Affiliations:** 1 Department of Gastroenterology and Metabolism, Nagoya City University Graduate School of Medical Sciences 158019 , Nagoya, Japan; 2 Advanced Life Science Institute Inc.Tokyo, Japan; 3 Department of Gastroenterology and Hepatology, Faculty of Life Sciences, Kumamoto University, Kumamoto, Japan; 4 Department of Clinical Laboratory Medicine, Nagoya City University Hospital 208519, Nagoya, Japan; 5 FUJIREBIO, INC.Tokyo, Japan; St. Jude Children's Research Hospital, Memphis, Tennessee, USA

**Keywords:** HBcrAg, HBcAg, phosphorylated HBcAg, CLEIA, HBV particles, clinical utility

## Abstract

To assess the performance and clinical relevance of three assays—hepatitis B core-related antigen (HBcrAg), hepatitis B core antigen (HBcAg), and phosphorylated HBcAg (pHBcAg)—in quantifying HBcrAg, a critical biomarker of hepatitis B virus (HBV) infection, fully automated chemiluminescent enzyme immunoassays (CLEIA) for two HBcAg variants were developed. Cutoff values for HBcAg and pHBcAg assays were established at 2.50 and 2.10 LogU/mL, respectively. The strong correlations among components (*r* = 0.896–0.970) were observed by using purchased 100 plasma samples. When the dynamics of HBcrAg components were analyzed by using a series of hepatitis B e antigen (HBeAg) seroconversion panel, alongside correlations with HBV markers, distinct dynamics and molecular profiles were observed. For the assessment of the clinical relevance of these assays, serum samples were obtained from 102 HBV-infected patients treated with nucleos(t)ide analog (NA). Both HBcAg and pHBcAg levels were significantly reduced during NA therapy, but in different patterns, suggesting HBV activity independent of HBV DNA and persistent HBcrAg components derived from pc-mRNA. OptiPrep density gradient ultracentrifugation analysis identified pHBcAg as the dominant component in high-density fractions. Immunoprecipitation and Western blotting confirmed that HBcAg components were predominantly enveloped by hepatitis B surface antigen (HBsAg), with pHBcAg identified as the major component of empty viral particles. Although the limitation in sample size in this study, the revealed distinct dynamics of HBcAg, pHBcAg, and HBcrAg during seroconversion and treatment suggest these assays could serve as independent biomarkers for monitoring intrahepatic HBV activity and treatment efficacy.

**IMPORTANCE:**

This study evaluates novel fully automated chemiluminescent enzyme immunoassay (CLEIA) systems for hepatitis B core antigen (HBcAg) and phosphorylated HBcAg (pHBcAg) and identifies pHBcAg as the predominant component of hepatitis B virus (HBV)-derived empty viral particles, challenging previous assumptions and providing new insights into HBV biomarkers. HBcAg and pHBcAg show distinct dynamics in hepatitis B e seroconversion and nucleos(t)ide treatment from the other biomarkers including hepatitis B core-related antigen (HBcrAg), HBV RNA, and HBV DNA. The developed CLEIA systems for HBcAg and pHBcAg show promise as tools for monitoring intrahepatic HBV activity, immune clearance, and noninfectious viral replication. Incorporating these biomarkers into clinical practice could refine HBV management strategies, improve reactivation risk prediction, and advance precision medicine approaches.

## INTRODUCTION

Chronic hepatitis B virus (HBV) infection remains a significant global health challenge despite advancements in antiviral therapies, including nucleos(t)ide analogs (NAs) and pegylated interferon-α. These treatments suppress viral replication and can achieve functional cure, marked by hepatitis B surface antigen (HBsAg) seroclearance (SC), thereby reducing the risks of cirrhosis and hepatocellular carcinoma (HCC) (1). However, HBsAg SC rates remain low (1), and recurrence frequently occurs after therapy due to the persistence of covalently closed circular DNA (cccDNA) in hepatocytes (2, 3). This cccDNA reservoir can reactivate viral replication, highlighting the need for innovative therapies and reliable biomarkers reflecting cccDNA activity.

Emerging HBV therapeutics, including antisense oligonucleotides, RNA interference agents, and capsid assembly modulators, offers potential to further suppress HBV replication (4–7). By targeting HBV RNA, these agents may enhance functional cure rates. As these therapies progress toward clinical application, sensitive and specific biomarkers that correlate with cccDNA activity are crucial for evaluating treatment efficacy and predicting long-term outcomes (8–11).

HBcrAg has been established as a surrogate marker for cccDNA activity, correlating with both serum HBV DNA and intrahepatic cccDNA levels (12–15). Derived from 3.5 kb precore mRNA (pc-mRNA) and pregenomic RNA (pgRNA), HBcrAg reflects the antigenic load of translated and processed products from these RNAs, which are transcribed from the cccDNA-associated precore promoter (PCP) and basal core promoter (BCP) regions. Hepatitis B e antigen (HBeAg) and p22cr (PreC) are translated from pc-mRNA which are designated as pc-mRNA derived polypeptide (pcRpp) in this paper, while HBcAg is produced from pgRNA (16). Using monoclonal antibodies (mAbs) targeting conserved polypeptide sequences in these antigens, the HBcrAg assay measures all these antigens (15).

Quantitative methods for measuring nonphosphorylated and phosphorylated hepatitis B core antigen (HBcAg and pHBcAg) have been reported (17–19). These assays provide quantitative measurements of HBcAg and pHBcAg, revealing distinct dynamics that correlate with HBV DNA and RNA levels. Analyses of natural infection and treatment cases suggest these novel biomarkers may complement existing markers, such as HBcrAg and HBeAg, but their clinical significance requires further investigation.

The phosphorylated C-terminal domain (CTD) of HBcAg plays a pivotal role in HBV replication by facilitating the encapsidation of pgRNA and reverse transcription to form infectious DNA particles (20–22). Phosphorylation of the CTD facilitates packaging of the pgRNA-polymerase complex, while coordinated CTD dephosphorylation and reverse transcription are required for the formation of infectious DNA particles. These particles are released into the bloodstream enveloped with HBsAg (20, 23, 24). Enveloped by HBsAg, these DNA particles are released into the bloodstream alongside RNA-containing and empty particles. Recent studies indicate that pHBcAg is the predominant protein in empty particles, challenging earlier assumptions that these particles primarily consist of p22cr (16, 25–27).

Building on the iTACT (immunoassay for total antigen including complex via pre-treatment)—HBcrAg chemiluminescent enzyme immunoassay (CLEIA) system, we developed fully automated CLEIA platforms for quantitatively measuring HBcAg and pHBcAg, using calibrators standardized by the HBcrAg CLEIA. These assays were validated and applied to plasma samples from HBV-infected individuals, including those previously analyzed. Additionally, we characterized HBcrAg components using OptiPrep density gradient ultracentrifugation. This study aims to elucidate the clinical significance of HBcAg and pHBcAg as biomarkers, offering potential advancements in HBV infection monitoring and personalized treatment strategies.

## MATERIALS AND METHODS

### Biological materials

Anti-CTD mAb HB50 (18) and anti-HBsAg mAbs HBs315 and HBs320 (28) were purified from hybridoma culture medium. Details on the establishment of anti-phosphorylated CTD are provided in the supplementary materials and methods (S-M&M). Recombinant ProHBeAg was expressed in *Escherichia coli* and purified as described (15). We used *E. coli*-expressed recombinant HBcAg and HBeAg, which are utilized for anti-HBcAg CLEIA and iTACT HBcrAg (FUJIREBIO, Tokyo, Japan), respectively. Plasma samples (*n* = 100) positive for HBsAg and HBV DNA were procured from TRINA BIOREACTIVES (Naenikon, Switzerland). The PHM935A/B HBV acute infection panels were purchased from Boston Biomedica Inc. (West Bridgewater, MA).

### Patients’ specimens

Serum samples were obtained from 102 HBV-infected patients treated with NA at Nagoya City University Hospital. Clinical parameters, including HBV-related ones, were measured during examinations between March 2022 and March 2024. Additionally, some of them who had stored sera over time and stored serum samples from before and after the administration of NA were used. All serum samples were stored at −80°C until analysis.

### iTACT-HBcrAg, HBcAg, pHBcAg CLEIA, and HBV biomarkers

The CLEIA method followed previously reported (29) with modifications (S-M&M). The procedures for other HBV biomarkers are described in S-M&M.

### OptiPrep density gradient ultracentrifugation analysis

OptiPrep [iodixanol: 60% (wt/vol) in water] was purchased from Serumwerk Bernburg (Bernburg, Germany). The procedure of ODG ultracentrifugation is described in S-M&M. Post-ultracentrifugation, 0.3 mL fractions were collected from the top of each tube, and the density was calculated based on weight measurements.

### Immunoprecipitation using anti-HBs mAbs

Magnetic particles immobilized with anti-HBsAg mAbs HBs315 and HBs320 were prepared following established protocols. Samples diluted with LUMIPULSE sample diluent (FUJIREBIO) were incubated with these magnetic particles overnight at 4°C. After removing unbound material, the particles were washed thrice with PBS (-). Captured components were released into a 2% SDS solution by incubating at 80°C for 2 min. The resulting supernatants were diluted 10-fold with the sample diluent and analyzed using iTACT-HBcrAg, HBcAg, and pHBcAg CLEIA. The details of the western blot analysis are described in S-M&M.

### Statistics

Statistical analyses were conducted using R (Version 4.4.1) (30) and Microsoft Excel (Microsoft Corporation, Redmond, Washington).

## RESULTS

### Sensitivity and specificity of the measurement system

**Fig 1:**
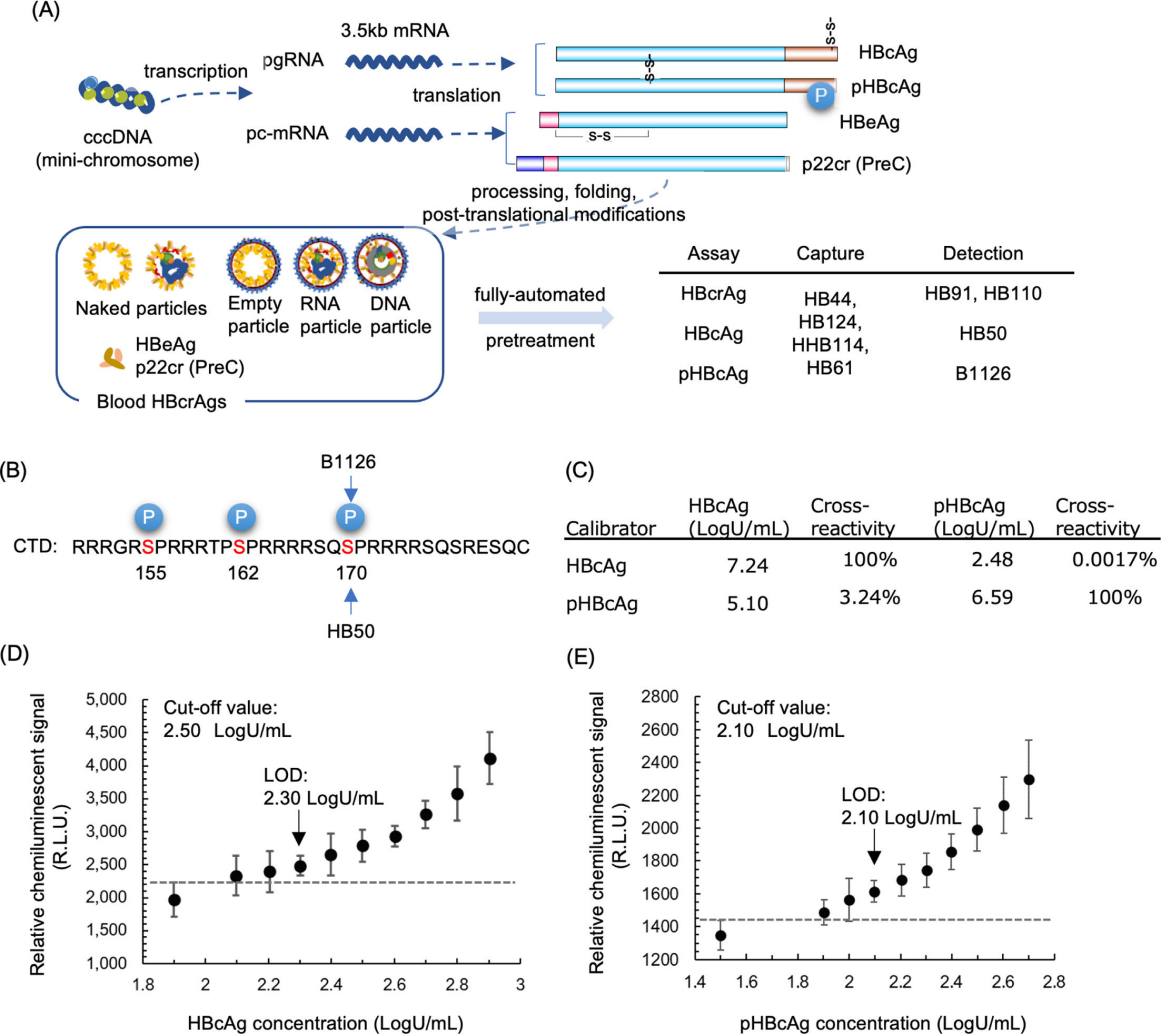
Configuration and performance of the three hepatitis B core-related antigen (HBcrAg) assays. (**A**) This schematic illustrates the expression of HBcrAg components from cccDNA and the combination of monoclonal antibody (mAb) for each iTACT-HBcrAg assay. HBcrAg components in blood are denatured in an acid-detergent solution and neutralized on the fully-automated chemiluminescent enzyme immunoassay (CLEIA) analyzer. The denatured antigens are captured by magnetic particles coated with monoclonal antibodies (mAbs) recognizing common HBcrAg polypeptide listed in the middle column in right side table (29). Captured antigens were probed with ALP-labeled monoclonal antibodies, which are listed in right column of the table, specific to hepatitis B core antigen (HBcAg), phosphorylated HBcAg (pHBcAg) and HBcrAg assays. Panel **B** summarizes the specific reactivity of HB50 and HB1126 to the phosphorylated serine residues within the c-terminal domain (CTD) of HBcAg. Panel **C** compares measurements of recombinant antigens used in HBcAg and pHBcAg assays using both systems. Panels **D** and **E** display the limits of detection (LOD) for iTACT-HBcAg and iTACT-pHBcAg, respectively, determined using serially diluted samples. Each concentration was measured in six replicates, and the mean values with standard deviations (SD) of the relative chemiluminescent signals (R.L.U.) are plotted.

The iTACT-HBcrAg, HBcAg, and pHBcAg assays used common procedures to capture total HBcrAg and measured HBcrAg, HBcAg, and pHBcAg using mAbs, HB91 and HB110, HB50 and B1126, respectively (Fig. 1A). The reactivity of HB50 and B1126 to synthetic peptides containing phosphorylated serine residues at various positions within the CTD suggested that HBcAg and pHBcAg could be distinguished by S170 phosphorylation in the CTD (Fig. 1B; Fig. S1a and b). The pHBcAg assay using B1126 was validated by the assay using A8115 mAb recognizing all three phosphorylated serine residues in the CTD (Fig. S1c and d). Measurement values for HBcAg and pHBcAg were standardized to the iTACT-HBcrAg assay and expressed in the same unit (LogU/mL). The cross-reactivity assessments of these assays are detailed in Fig. 1C.

The HBcAg and pHBcAg CLEIA detection and quantification limits were 2.30 LogU/mL and 2.10 LogU/mL (Fig. 1D and E), and 2.50 LogU/mL and 2.10 LogU/mL (not shown), respectively. Analysis of 100 HBV-negative specimens (not shown) set the HBcAg and pHBcAg CLEIA cutoffs at 2.50 LogU/mL and 2.10 LogU/mL, respectively.

### Correlations and ratios between the HBcrAg components and other HBV markers

**Fig 2:**
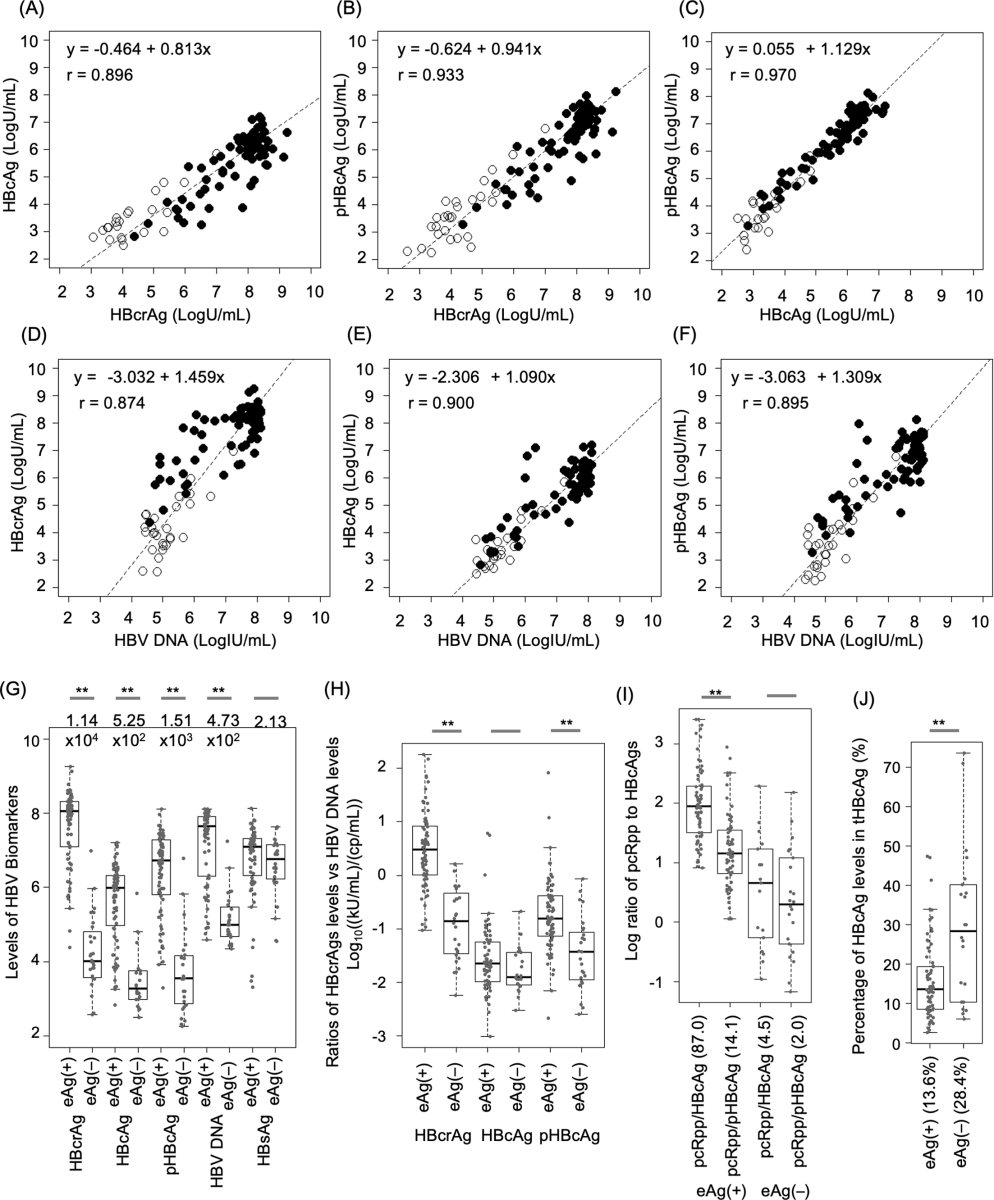
Comparison of hepatitis B core-related antigen (HBcrAg), hepatitis B core antigen (HBcAg), and phosphorylated HBcAg (pHBcAg) levels with each other or with hepatitis B virus (HBV) DNA levels of 100 purchased HBsAg positive plasma specimens. The upper panels show correlations between each HBcrAg level: HBcAg vs HBcrAg (**A**), pHBcAg vs HBcrAg (**B**), and pHBcAg vs HBcAg (**C**). The lower panels show correlations between each HBcrAg levels—HBcrAg (**D**), HBcAg (**E**), and pHBcAg (**F**)—and HBV DNA levels. HBcrAg and HBV DNA levels are shown logarithmically, with Passing-Bablok regression equations and Pearson correlation coefficients in each panel. Closed circles represent eAg(+) cases, while open circles represent eAg(–) cases. (**G**) Comparison of HBV biomarker levels between eAg(+) and eAg(–) cases. These levels are visualized as box plots on a log_10_ scale. The measurement units are U/mL for HBcrAg, HBcAg, and pHBcAg and IU/mL for HBV DNA and HBsAg. Bars with double asterisks indicate differences in HBV biomarker levels between eAg(+) and eAg(–) cases with *P*-values under 0.05 as determined by the Wilcoxon signed rank test. The numbers under the bars indicate the ratios of the median values of each antigen level in eAg(+) cases to those in eAg(–) cases. (**H**) Box plots show the ratios of HBcrAg, HBcAg, and pHBcAg to HBV DNA levels expressed as log_10_[(kU/mL)/(cp/mL)]. Bars with double asterisks indicate differences in ratios between eAg(+) and eAg(–) cases with *P*-values under 0.05 by the Wilcoxon signed rank test. (**I**) Box plots show ratios of precore mRNA-derived polypeptide (pcRpp) levels to hepatitis B core antigen (HBcAg) and phosphorylated HBcAg (pHBcAg) levels. The levels of pcRpp represent the levels of HBcrAg with the total HBcAg (tHBcAg) levels subtracted from it. The tHBcAg levels are the sums of the HBcAg and pHBcAg levels. These ratios are expressed on a log_10_ scale. Numbers in parentheses are the median values of the ratios. Bars with double asterisks indicate differences between HBcAg ratio and pHBcAg ratio with *P*-values under 0.05 as determined by the Wilcoxon signed rank test. (**J**) Box plots show the percentage of HBcAg within tHBcAg. Bars with double asterisks indicate differences in percentage between eAg(+) and eAg(–) cases with *P*-values under 0.05 as determined by the Wilcoxon signed rank test. Numbers in parentheses are the median values of percentages.

We analyzed 100 plasma samples that were positive for HBsAg and HBV DNA, predominantly from male donors. The HBV genotypes were distributed as follows: A:B:C:D:E = 29:35:24:11:1. These samples were divided into two groups: 72 HBeAg-positive [eAg(+)] and 28 HBeAg-negative [eAg(–)] (Table S1). The median age of the eAg(+) cases was 21 years, approximately 20 years younger than the eAg(–) cases, reflecting a significant difference in genotype B (Fig. S2a). Significant differences in median ages were found between the genotypes (Fig. S2b). Despite these biases, HBcrAg components strongly correlated with one another (*r* = 0.896–0.970) and with HBV DNA (*r* = 0.874–0.900) (Fig. 2A through F) regardless of the HBeAg status (Table S2) and genotypes (not shown).

Among the quantitative HBV biomarkers, HBcrAg, HBcAg, pHBcAg, and HBV DNA levels were significantly lower in eAg(–) cases than in eAg(+) cases to different degrees (4.73 × 10^2^–1.14 × 10^4^) (Fig. 2G). While the HBcAg-to-HBV DNA ratio was similar between eAg(+) and eAg(–) cases, consistent with the fact that HBcAg constitutes the capsids of DNA particles, both the HBcrAg- and pHBcAg-to-HBV DNA ratios differed significantly between these groups (Fig. 2H).

In eAg(+), the levels of pcRpp—calculated by subtracting the sum of HBcAg and pHBcAg from the HBcrAg level—were 87-fold higher (median) than HBcAg and 14.1-fold higher (median) than pHBcAg (Fig. 2I). These ratios were higher in genotype C (GenC) than in genotype A (GenA) and genotype B (GenB) (Fig. S3). In eAg(–) cases, the ratios of pcRpp-to-HBcAg and pHBcAg were 4.5 and 2.0, respectively, suggesting the presence of pcRpp in most eAg(–) cases (Fig. 2I). The percentages of HBcAg within tHBcAg were significantly different between eAg(+) and eAg(–) cases (Fig. 2J), suggesting that HBcAg and pHBcAg, which are derived from pgRNA, would be distinctive biomarkers with different properties over the course of HBeAg seroconversion.

### Dynamics and characterization of HBV biomarkers derived from cccDNA in the HBe seroconversion panel

**Fig 3:**
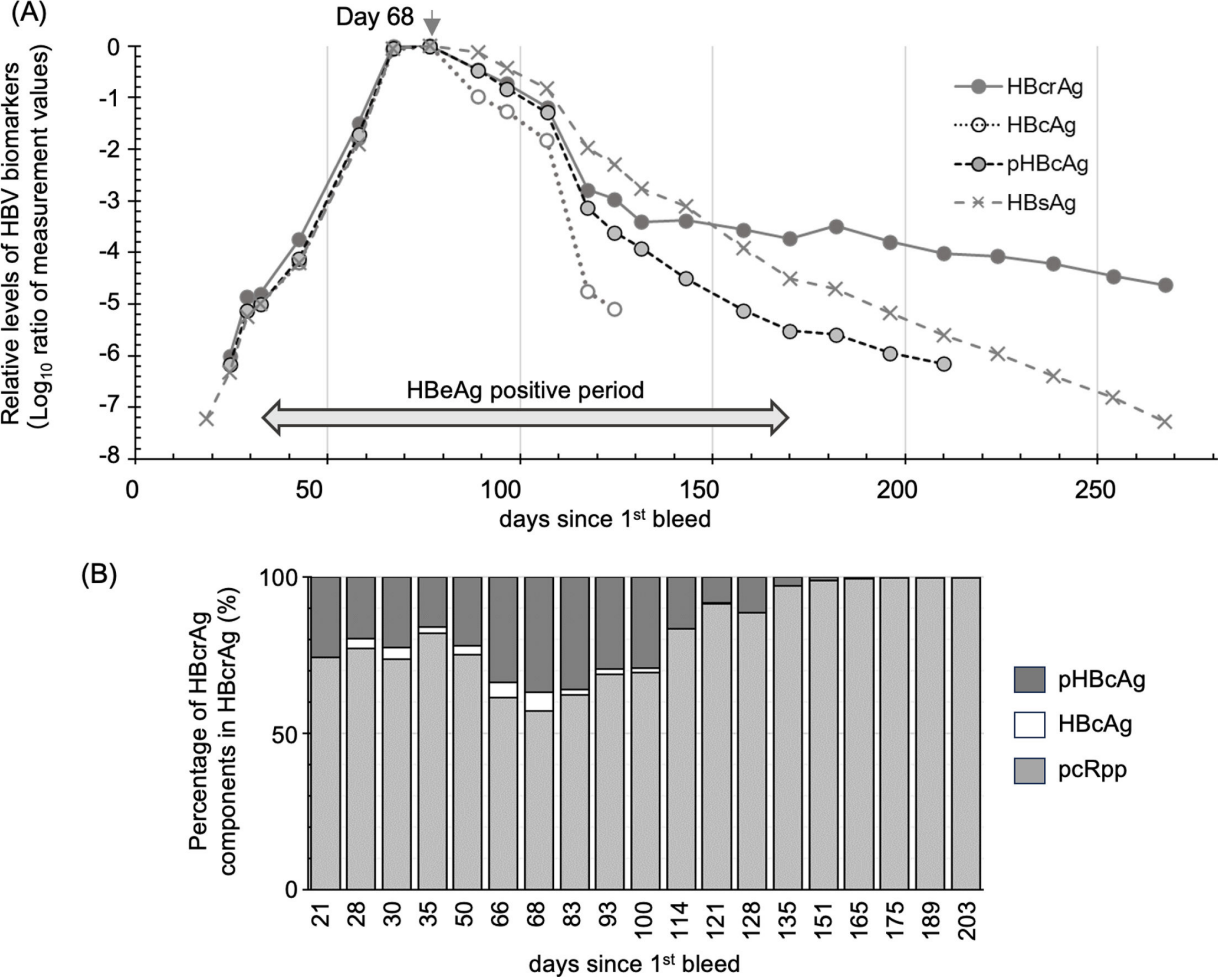
Dynamics of quantitative hepatitis B virus (HBV) biomarkers during HBe seroconversion of PHM935A and B panels. Panel **A** shows relative levels of hepatitis B core-related antigen (HBcrAg), hepatitis B core antigen (HBcAg), phosphorylated HBcAg (pHBcAg), and high-sensitivity hepatitis surface antigen (HBsAg) compared to those of the peak point, day 68 indicated by the down arrow. The values are expressed in log_10_ ratios of measurement values. The gray double arrow marks the HBeAg-positive period. HBeAb and HBsAb became positive on days 246 and 231, respectively (Table S3). Values below each assay’s cutoff are excluded from the plot. The horizontal axis shows collection days from the first bleed, with markers and lines for values in panel. Panel **B**, bar plot, shows percentage of HBcAg, pHBcAg, and pc-mRNA derived polypeptide (pcRpp) in HBcrAg at the collecting point since first bleed.

We analyzed the dynamics of HBcrAg, HBcAg, and pHBcAg during acute HBeAg-HBeAb seroconversion using panels PHM935A/B (Fig. 3A; Table S3). The dynamics of these antigens aligned with that described previously (19) and peaked on day 68 since the first bleed. The composition of the HBcrAg components changed over the course of HBeAg seroconversion. The percentages of HBcAg and pHBcAg within HBcrAg peaked on day 68 and declined at different rates thereafter (Fig. 3A and B), resulting in pcRpp becoming the major component of HBcrAg after HBeAg seroconversion.

**Fig 4:**
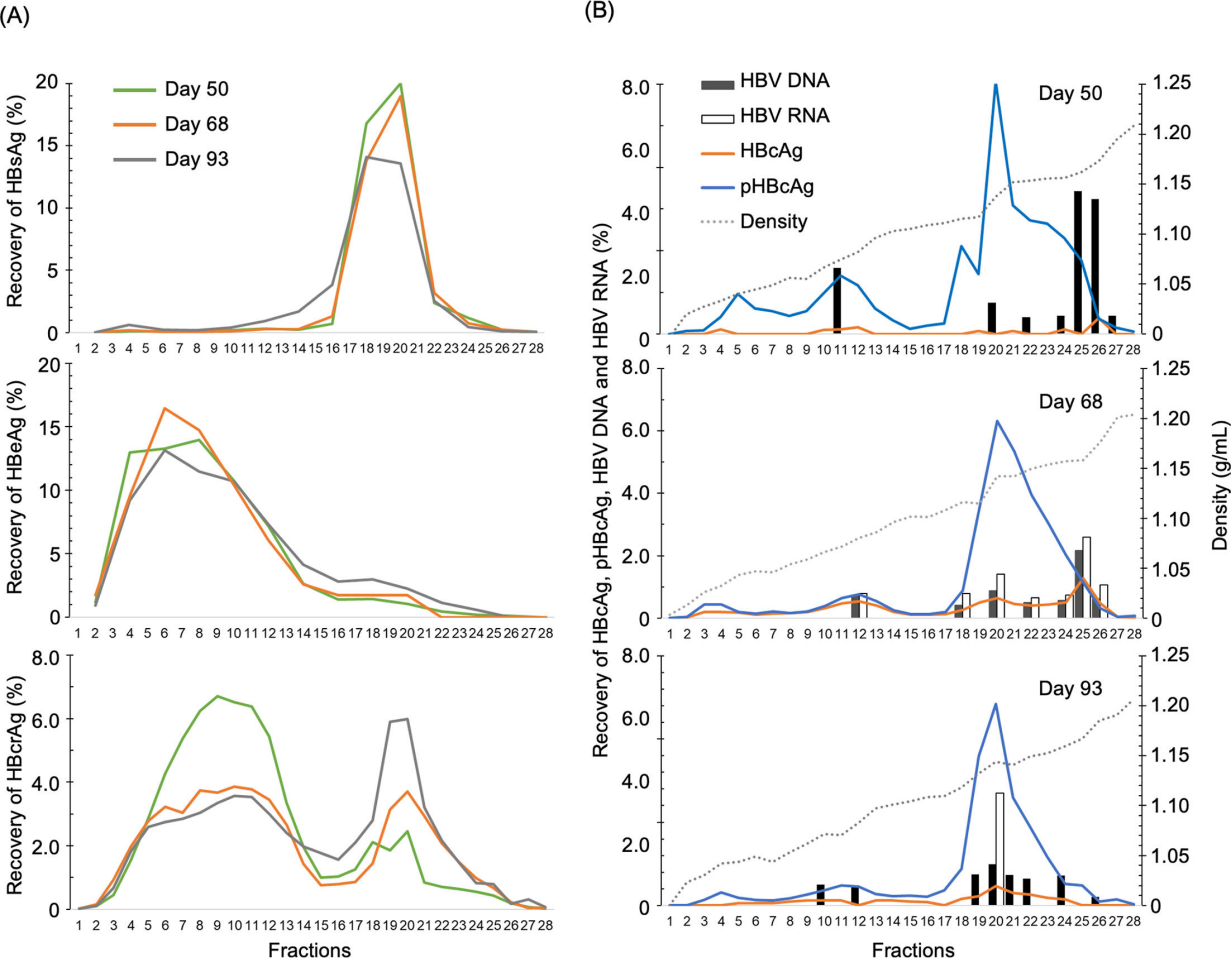
Fractionation of hepatitis B virus (HBV) biomarkers in day 50, day 68, and day 93 samples from the PHM935A seroconversion panel using OptiPrep density gradient (ODG) ultracentrifugation. (**A**) Panels illustrate the fractionation of HBV biomarkers: the top, middle, and bottom panels indicate the recovery rates for hepatitis surface antigen (HBsAg), hepatitis e antigen (HBeAg), and hepatitis B core-related antigen (HBcrAg), respectively. (**B**) Panels display the recovery rates of hepatitis core antigen (HBcAg), phosphorylated HBcAg (pHBcAg), and HBV DNA, HBV RNA of day 50 (top), day 68 (middle), and day 93 (bottom). Their recovery rates in percentage are indicated on the left axis. The density of the fractions is indicated on the right axis. The horizontal axes of all panels indicate the fraction numbers of the ODG ultracentrifugation analysis. The marks of each panel are shown at the inlets of the top panels.

We characterized the HBcrAg components in samples on days 50, 68, and 93 from the PHM935A seroconversion panel using ODG ultracentrifugation analysis (Fig. 4). The peaking patterns of HBsAg [Fractions (Fr)18-21] and the widely distributed fractionation patterns of HBeAg (Fr4–12) were very similar at the three observation points. In contrast, an increase in the proportion of high-density fractions (Fr18–26) was observed in the fractionation pattern of HBcrAg over the observation period (Fig. 4A). The fraction patterns of HBcrAg in the high-density fractions (Fr18–26) resembled those of pHBcAg, shifting toward the low-density side (Fr20–22) over the observation period. The fraction patterns of HBcAg, HBV DNA, and HBV RNA, which peaked in fractions from 24 to 26 on days 50 and 68, shifted to the lower-density fractions (Fr20–22) on day 93 (Fig. 4B). These data suggested not only quantitative but also qualitative changes in the HBcrAg components during HBeAg seroconversion.

### Analysis of HBcrAg components during the NA treatment

**Fig 5:**
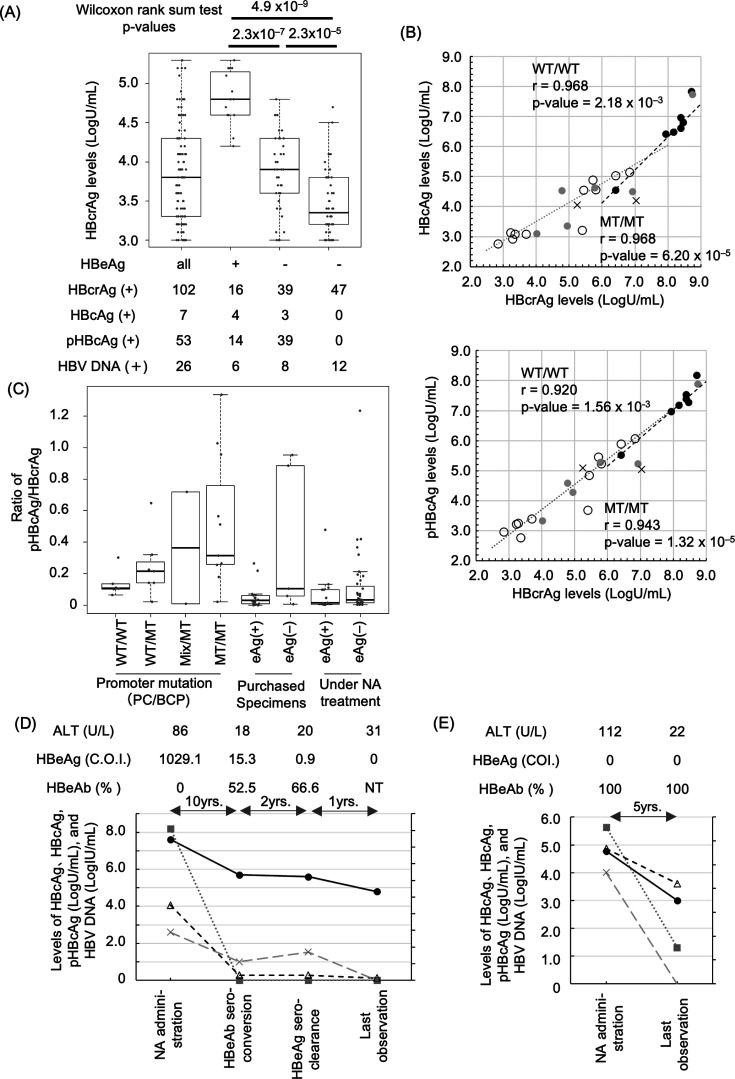
Evaluation of hepatitis B core antigen (HBcAg) and phosphorylated HBcAg (pHBcAg) using clinical specimens collected from patients with nucleos(t)ide analog (NA) treatment. (**A**) Comparison of hepatitis B core-related antigen (HBcrAg) levels in patient serum samples with NA treatment. HBcrAg-positive cases (*n* = 102) with NA treatment were grouped based on hepatitis B e antigen (HBeAg) and pHBcAg status. HBcrAg levels for each group are presented as box plots. Significant differences (Wilcoxon rank-sum test, *P* < 0.05) between groups are indicated by bars and p-values above the plots. The number of HBcAg-, pHBcAg-, and HBV DNA-positive cases within each group is shown below the plot. (**B**) Correlations between HBcrAg levels (horizontal axis) and HBcAg levels (vertical axis) (upper panel), and pHBcAg levels (vertical axis) (lower panel) at NA administration points are depicted for groups with precore (PC)/ basal core promoter (BCP) sequences classified as wildtype (WT)/WT (*n* = 7, black closed circles), WT/mutant type (MT) (*n* = 6, gray closed circles), WT and MT mixed quasispecies (Mix)/MT (*n* = 2, black crosses), and MT/MT (*n* = 11, black open circles). Passing Bablok regression curves for WT/WT cases and MT/MT cases are shown in black-dashed lines and gray-solid lines, respectively. Pearson correlation coefficient and *P*-values of WT/WT and MT/MT for HBcAg or pHBcAg to HBcrAg are indicated in inlets. (**C**) A box plot illustrates the ratios of HBcrAg to HBcAg levels across WT/WT, WT/MT, Mix/MT, and MT/MT groups, as well as NA-treated and purchased specimen groups. Statistical comparisons using the Wilcoxon rank-sum test are summarized in Table 2. Biomarker kinetics during NA treatment are shown for an eAg(+) case (**D**) and an eAg(–) case (**E**). HBcrAg levels (black lines and closed circles), HBV DNA levels (gray dotted lines and squares), HBcAg/tHBcAg ratios (long gray dashed line and cross marks), and pHBcAg/HBcrAg ratios (black dashed line and open triangles) are plotted. ALT levels, HBeAg values (cut-off index: C.O.I.), and HBeAb percent inhibition are displayed above the plots, with double-headed arrows indicating the time intervals (years) between clinical observation points.

Because the clinical background of the purchased specimens was unclear, we further analyzed the HBcrAg components in the NA-treated clinical cases. We classified serum samples from 102 NA-treated patients with positive HBcrAg based on HBeAg status (Table 1) and compared pHBcAg detection rates. Approximately 88% (14/16) of eAg(+) samples were measurable for pHBcAg, while only 45% (39/86) of eAg(–) samples were detectable (Fig. 5A). Among quantifiable HBcAg samples, 25% (4/16) were eAg(+), compared to 3.5% (3/86) for eAg(–). HBV DNA was undetectable in approximately three-quarters of eAg(–) patients, with no significant differences between pHBcAg, HBcAg-positive and -negative cases (not shown), indicating that pHBcAg and HBcAg are independent of HBV DNA. In addition, all cases in which HBV DNA was detected were also detected below quantitation due to NA treatment. In the eAg(–) group, pHBcAg-positive cases exhibited significantly higher HBcrAg levels (Fig. 5A *P*-value = 2.3 × 10^–5^). Sensitivity for HBcAg and pHBcAg was particularly low in treated eAg(–) patients, potentially reflecting levels below detection thresholds due to ongoing treatment.

**TABLE 1:** Summary of 102 cases collected from patients with NA treatment^*c*^

Characteristic (*n* = 102)	eAg(+) (*n* = 16)	eAg(–) (*n* = 86)	*P*-value^*b*^
Gender, male/female	8/8	57/29	0.261
Age, years^*a*^	47 (41‒51)	54 (47‒61)	0.140
HBV genotype, A/B/C/N.T.	0/1/15/0	2/8/61/15	0.226
Platelet (×10^3^/µL)^*a*^	217 (196‒246)	221 (177‒267)	0.923
AST (U/L)^*a*^^,^^*c*^	21 (19‒22)	22 (19‒26)	0.501
ALT (U/L)^*a*^^,*c*^	22 (16‒24)	19 (15‒26)	0.775
FIB4 index^*a*^	0.92 (0.77‒1.53)	1.22 (0.86‒1.79)	0.230
HBsAg (IU/mL)^*a*^	796.58 (355.12‒3,125.31)	459.38 (118.57‒1,228.08)	0.131
HBcrAg (logU/L)^*a*^	4.8 (4.6‒5.1)	3.6 (3.2‒4.0)	<0.0001
HBV DNA, quantitative/not quantitative but detect/not detect	0/6/10	0/20/66	0.230
Period from NA administration to serum sample collection, months^*a*^	88 (64‒151)	146 (82‒188)	0.024
Type of NA: ETV/TDF/TAF/LAM + TAF/ETV + TAF	1/0/14/0/1	32/2/44/4/4	0.060

^*a*^ Data from all patients are expressed as numbers for categorical data and medians (first–third quartiles) for non-categorical data.

^*b*^ Categorical variables were compared between groups using the chi-square test, and non-categorical variables were compared using the Mann-Whitney *U* test.

^*c*^ ALT, alanine aminotransferase; AST, aspartate transferase.

We assessed the impact of PC and BCP mutations on the correlation between HBcAg, pHBcAg, and HBcrAg levels using baseline pre-treatment samples (Table S4). Among 6 cases with wild-type (WT)/WT PC/BCP mutations, 5 were HBeAg-positive, while 9 of 11 mutation type (MT)/MT cases were eAg(–). Regardless of mutation status, HBcrAg, HBcAg, and pHBcAg levels were significantly correlated (Fig. 5B). The pHBcAg/HBcrAg ratio was significantly higher in MT/MT than WT/WT cases (Table 2).

**TABLE 2:** Comparison of the pHBcAg/HBcrAg ratios among specimens by Wilcoxon rank sum test^*a*^

		PC/BCP mutation status			HBeAg status of purchased specimen		HBeAg status of cases under NA treatment	
		WT/MT	Mix/MT	MT/MT	eAg (+)	eAg(–)	eAg (+)	eAg(–)
PC/BCP mutation status	WT/WT	1.34 × 10^–1^	1.00	**2.37 × 10^–2^**	**3.79 × 10^–3^**	1.00	**2.88 × 10^–3^**	6.88 × 10^–2^
	WT/MT		1.00	1.03 × 10^–1^	**6.59 × 10^–3^**	8.71 × 10^–1^	**5.15 × 10^–3^**	**1.57 × 10^–2^**
	Mix/MT			6.22 × 10^–1^	5.90 × 10^–1^	8.46 × 10^–1^	4.75 × 10^–1^	8.32 × 10^–1^
	MT/MT				**1.80 × 10^–4^**	3.08 × 10^–1^	**3.40 × 10^–4^**	**2.20 × 10^–4^**
HBeAg status of purchased specimen	eAg (+)					8.80 × 10^–2^	9.27 × 10^–1^	2.60 × 10^–1^
	eAg(–)						1.51 × 10^–1^	2.09 × 10^–1^
HBeAg status of cases under NA treatment	eAg (+)							2.00 × 10^–1^

^*a*^ Bold letters indicate *P*-values less than 0.05.

Comparing pHBcAg/HBcrAg ratios between 100 commercially purchased samples and 102 NA-treated patients, the ratio for WT/WT cases, predominantly from eAg(+) cases, was significantly higher than those in both commercial and NA-treated cohorts. Conversely, the ratio for treated eAg(–) cases was significantly lower than treatment-naive or purchased samples at initiation (Fig. 5C, Table 2), suggesting NA treatment may alter the pHBcAg/HBcrAg ratio.

We tracked HBcrAg, HBcAg, and pHBcAg dynamics in two NA-treated cases with serial analysis over time. Case 1, a 60-year-old man, had HBeAg levels of 1,029.1 cut-off index with WT/MT of PC/BCP mutations, HBV DNA of 8.2 log IU/mL, and ALT of 83 U/L and was treated with entecavir. HBV DNA became undetectable, and ALT normalized during treatment. Both the pHBcAg/HBcrAg and HBcAg/tHBcAg ratios decreased after NA initiation (Fig. 5D). Case 2, a 38-year-old man, HBeAg-negative with WT/MT of PC/BCP mutations, HBV DNA of 5.6 log IU/mL, and ALT of 112 U/L, was treated with tenofovir alafenamide. Post-treatment, HBV DNA became undetectable, ALT normalized, and both the pHBcAg/HBcrAg and HBcAg/tHBcAg ratios declined similar to Case 1 (Fig. 5E). These data indicated that pHBcAg and pcRpp dynamics were different from those of other cccDNA surrogate biomarkers as HBcAg and HBV DNA during the NA-treatment.

### Almost all HBcAg and pHBcAg are enveloped with HBsAg

**Fig 6:**
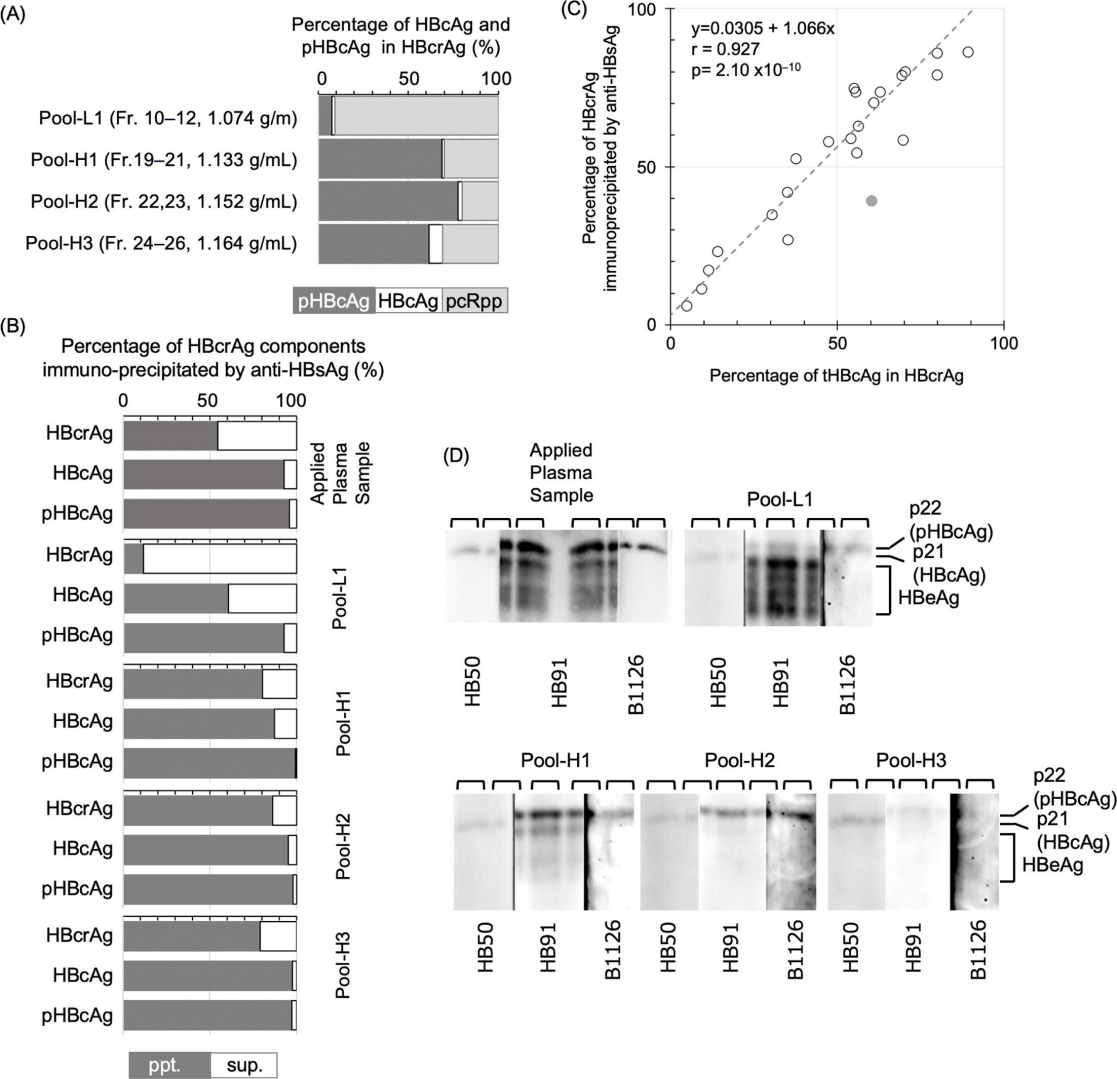
Analysis of the enveloped hepatitis B core-related antigen (HBcrAg) components fractionated by OptiPrep density gradient (ODG) ultracentrifugation. (**A**) The percentages of hepatitis B core antigen (HBcAg), phosphorylated HBcAg (pHBcAg), and precore mRNA derived polypeptide (pcRpp) in HBcrAg for the pooled fractions of ODG ultracentrifugation for the PHM935A day-68 sample. The fraction pool samples analyzed are indicated at the left side of the panel with their fraction numbers and average density of each fraction pool. The dark gray, white, and light gray bars indicate percentage of pHBcAg, HBcAg, and pcRpp, respectively. (**B**) The percentages of HBcrAg, HBcAg, and pHBcAg immunoprecipitated by anti-HBsAg for the pooled fractions of ODG ultracentrifugation for the PHM935A day-68 sample as shown in panel **A**. The samples analyzed are indicated at the right side of each panel. (**C**) Correlation analysis of tHBcAg and anti-HBsAg immunoprecipitated HBcrAg percentages in fraction pools from density gradient ultracentrifugation analyses conducted on day 50, day 68, and day 93 of PHM935A panel, HBV220, and HBV249 is summarized (Fig. S5 and Table S6). The Passing Bablok regression equation and Pearson correlation coefficient are provided in the inlet, with gray circles representing pool-H1 values from the HBV220 analysis. (**D**) Western blot (WB) analysis of the PHM935A day 68 sample as shown in panel **A**. The samples analyzed are indicated at the top of each panel. The lane positions were indicated above panels. The stripped blot was probed with monoclonal antibodies listed at the bottom of each panel, with HBcrAg component positions marked on the right.

We analyzed the proportions of enveloped pHBcAg and HBcAg in the pooled fractions of ODG ultracentrifugation analysis (Fig. 4, Fig. S4) using immunoprecipitation with anti-HBsAg mAbs (Fig. 6; Fig. S5, Table S5 and S6). The percentages of HBcrAg immunoprecipitated by the HBsAg antibody closely correlated with those of tHBcAg in HBcrAg, with nearly all pHBcAg and HBcAg recovered in the precipitate (Fig. 6 through C; Fig. S5), except for specific pooled fractions with poor HBcAg recovery: Pool-H1, which corresponded to average density of 1.135 g/mL, of day 50, Pool-L1 (1.084 g/mL) and H3 (1.171 g/mL) of day 93, and Pool-H1 (1.147 g/mL) and H2 (1.148 g/mL) of HBV220. These results indicate that these fractions likely contain naked-capsid particles or immune complexes with hepatitis B core antibody not enveloped by HBsAg (31) and that pHBcAg is the predominant component of enveloped HBcrAg in most cases.

Pooled fractions from Day 68 of PHM935A were also analyzed by Western blot using anti-HBcrAg mAbs, HB91, HB50, and B1126, which are detector mAbs for HBcrAg, HBcAg, and pHBcAg, respectively. In the plasma sample, the HB91-detected bands corresponded to HBeAg molecules with heterogeneously processed CTD in GenA (16). A higher molecular weight band identified by HB91 matched the band detected by B1126, indicating the presence of pHBcAg. Additionally, a faint HB50-detected band, representing nonphosphorylated HBcAg (p21), was observed between the pHBcAg and HBeAg bands, as previously (26). Given the densities of Pool-H1 (1.133 g/mL) and H2 (1.152 g/mL) matched those analyzed in the prior study, we infer that the majority of what was identified as p22cr in that report corresponds to pHBcAg (Fig. 6D).

## DISCUSSION

In this study, we developed a fully automated CLEIA system for quantifying HBcAg and pHBcAg, leveraging the iTACT-HBcrAg CLEIA platform. Using calibrators standardized to the HBcrAg CLEIA, our system provides precise quantification of HBcrAg components, revealing distinct roles for HBcAg and pHBcAg in HBV management. Crucially, our findings challenge previous assumptions about the composition of empty viral particles. Earlier studies, relying on HB50 reactivity, attributed p22cr as the primary component (26). However, we demonstrate that HB50 does not recognize the dominant S170-phosphorylated CTD, confirming that pHBcAg is the principal component of HBcrAg in these fractions. While trace amounts of p22cr(PreC) cannot be entirely excluded, our results align with recent findings identifying pHBcAg as the primary component of empty particles (16, 27). This redefinition of particle composition suggests that HBcrAg detected before HBV reactivation is predominantly pHBcAg (32), providing new insights into HBV reactivation and its biomarkers.

In HBeAg-negative acute and chronic hepatitis B, while HBeAg levels decline, HBcrAg can remain elevated. The predominant component of persistent HBcrAg is likely pcRpp, which may serve as a marker of HBV replication activity and persistent infection. Given its ability to reflect ongoing viral transcription and replication, HBcrAg quantification, particularly through the differentiation of its components, could provide valuable insights for clinical decision-making. This assay system offers a potential tool for assessing HBV persistence and predicting treatment responses, particularly in patients undergoing antiviral therapy or functional cure strategies. The ability to distinguish pHBcAg from HBcAg further enhances its utility in monitoring disease progression and treatment efficacy.

HBcrAg uniquely captures multiple aspects of the HBV life cycle, including cccDNA activity, RNA transcription, and viral particle production. It reflects the antigenic load of translated and processed products from both pgRNA and pc-mRNA, encompassing pcRpp composed of HBeAg and p22cr(PreC), HBcAg in DNA-containing particles, and pHBcAg in empty or RNA-containing particles. This broad scope makes HBcrAg a dynamic marker for tracking ongoing viral replication and transcription. Accurately measuring these components is essential for understanding the progression of HBV infection and evaluating therapeutic efficacy, particularly in the context of functional cure strategies targeting HBsAg SC.

Our findings underscore the need for further validation of HBcAg and pHBcAg CLEIA systems across diverse patient populations. Factors such as genotype, natural history, treatment history, and mutation bias may influence the ratios of HBcrAg components, underscoring the importance of personalized approaches in HBV management. Additionally, the limited analysis of both eAg(+) and eAg(–) cases in this study warrants broader investigations to fully elucidate the differential roles of these biomarkers. Large-scale cohort studies are essential to explore the natural and treatment history of hepatitis B and its links to hepatocarcinogenesis. Furthermore, the correlations of pHBcAg and pcRpp to intrahepatic cccDNA should be addressed. To address these issues, more sensitive and scalable measurement systems will maximize the potential of HBcAg and pHBcAg as precision medicine tools.

In summary, our study redefines the composition of empty viral particles, establishing pHBcAg as the predominant component and challenging long-standing assumptions. These findings emphasize the critical role of pHBcAg, HBcAg and HBcrAg representing pcRpp as biomarkers for monitoring cccDNA activity, capturing noninfectious viral dynamics, and optimizing HBV treatment strategies including emerging therapeutic strategies targeting intrahepatic HBV activity. Although further validation in large cohorts is required, integrating these biomarkers into clinical practice has the potential to refine therapeutic approaches, improve patient outcomes, and advance HBV management.

## SUPPLEMENTAL MATERIAL

Supplemental material jcm.00385-25-s0001.docx Supplemental methods, Fig. S1 to S5, and Tables S1 to S6.

ASM does not own the copyrights to Supplemental Material that may be linked to, or accessed through, an article. The authors have granted ASM a non-exclusive, world-wide license to publish the Supplemental Material files. Please contact the corresponding author directly for reuse.
