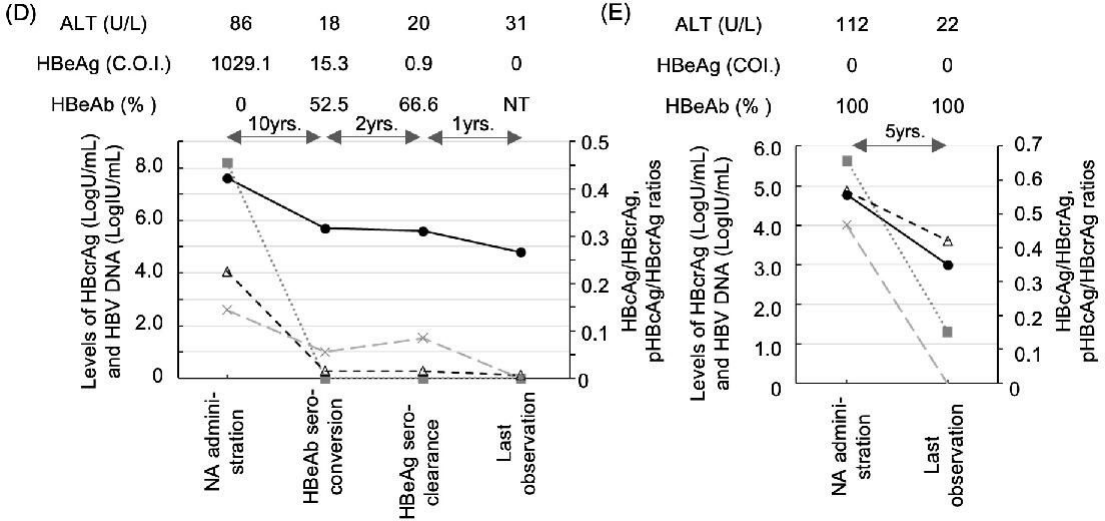# Correction for Suzuki et al., “Evaluation of fully automated chemiluminescent enzyme immunoassays for hepatitis B core-related antigen components, phosphorylated and non-phosphorylated hepatitis B core antigens: clinical significance and dynamics during hepatitis B e antigen seroconversion”

**DOI:** 10.1128/jcm.01465-25

**Published:** 2026-02-18

**Authors:** Takanori Suzuki, Chiharu Ohue, Osamu Arai, Yuka Inose, Katsuya Nagaoka, Shintaro Ogawa, Takako Inoue, Kentaro Matsuura, Katsumi Aoyagi, Shintaro Yagi, Yasuhito Tanaka

## AUTHOR CORRECTION

Volume 63, no. 9, e00385-25, 2025, https://doi.org/10.1128/jcm.00385-25. In Fig. 1A, “HHB114” should read “HB114”.

In Fig. 6A, “1.074 g/m” should be “1.074 g/mL”.

Figure 5D and E should appear as shown in this correction. These corrections do not affect the overall conclusions of the study.

**Fig 5:**