# Supplementary material for: Evaluation of fully automated chemiluminescent enzyme immunoassays for hepatitis B core-related antigen components, phosphorylated and non-phosphorylated hepatitis B core antigens: clinical significance and dynamics during hepatitis B e antigen seroconversion
Source: J Clin Microbiol. 2025 Aug 19;63(9):e00385-25. doi: 10.1128/jcm.00385-25 (PMC12421862; doi:10.1128/jcm.00385-25)
Supplement: Supplemental material — Supplemental methods, Fig. S1 to S5, and Tables S1 to S6. [file jcm.00385-25-s0001.docx]

**Supplementary Materials**

**Evaluation of Fully Automated Chemiluminescent Enzyme Immunoassays for Hepatitis B core-Related Antigen Components, Phosphorylated and Non-Phosphorylated Hepatitis B Core Antigens: Clinical Significance and Dynamics During Hepatitis B e Antigen Seroconversion.**

Takanori Suzuki, Chiharu Ohue, Osamu Arai, Yuka Inose, Katsuya Nagaoka, Shintaro Ogawa, Takako Inoue, Kentaro Matsuura, Katsumi Aoyagi, Shintaro Yagi, Yasuhito Tanaka

Supplementary Materials and Methods p2

Supplementary Figures p7

Supplementary Tables p14

Supplementary References p20

**Supplementary materials and methods**

HBV biomarkers

The HBV genotypes of 100 purchased plasma specimens were determined via EIA using mAbs targeting the PreS2 epitope (1) (IMMUNIS HBV genotype, Institute of Immunology Co., Ltd., Tokyo, Japan). Specimens with indeterminate EIA results were genotyped using the Invader-PCR method at BML, Kawagoe, Japan (2). HBeAg, HBeAb, and HBsAg levels were measured with LUMIPULSE PRESTO HBeAg, HBeAb, and HBsAg-HQ assays (FUJIREBIO, Tokyo) using the fully automated analyzer (Lumipulse L2400, FUJIREBIO, Tokyo). HBV RNA and DNA titers were quantified using cobas HBV RNA (RUO) and cobas HBV on a Cobas 8800 (ROCHE, USA) at SRL, Akiruno, Japan.

Establishment of antiphosphorylated CTD monoclonal antibodies

The peptide RRRGR [pS] PRRRTP [pS] PRRRRSQ [pS] PRRRRSQSRESQC conjugated to Imject™ Maleimide-Activated BSA (Thermo Scientific™) or the peptide CRRRGRSPRRRTPSPRRRRSQ [pS] PRRRRSQSRESQ conjugated to Imject™ Maleimide-Activated mcKLH (Thermo Scientific™) was diluted to 1.0 mg/ml in 10 mM phosphate buffer (pH 7.3) with 0.15 M NaCl (PBS). This solution was emulsified with Freund’s adjuvant, and 50–100 μg was subcutaneously administered to 46-week-old BALB/c mice. Booster immunizations were given biweekly, with a final intraperitoneal administration of 100 μg antigen protein in PBS.

Three days post-final immunization, the spleen was aseptically removed from the mouse and disaggregated into single cells using tweezers and a cell strainer, followed by suspension in serum-free RPMI1640 medium. The cells were pelleted by centrifugation (1000 rpm, 5 min, room temperature), treated with 1 ml of Red Blood Cell Lysing Buffer (MERCK), and left at room temperature for 1 min. Subsequently, 20 mL of serum-free RPMI1640 medium was added, and the cells were resuspended thoroughly. The cell pellet was re-obtained via centrifugation (1000 rpm, 5 min, room temperature) and resuspended in serum-free RPMI1640 medium. Concurrently, myeloma cells (mouse myeloma cell line Sp2/0 Ag14) in the logarithmic growth phase were collected, centrifuged (1000 rpm, 5 min, room temperature), and resuspended in serum-free RPMI1640 medium. Spleen and myeloma cells were mixed at a 1:1 ratio to achieve a total cell count of 3.4 ×10^7^. The cell mixture underwent centrifugation (1000 rpm, 5 min, room temperature), and the supernatant was discarded. The cells were washed twice with ECF buffer (0.3 M mannitol, 0.1 mM calcium chloride, 0.1 mM magnesium chloride solution). The cell mixture, suspended in 0.35 mL of ECF buffer, was subjected to cell fusion using a cell fusion device (ECFG21, Neppa Gene Co., Ltd.) and a platinum electrode (MS stand-type chamber, CUY497P2, 0.8 mL) under these conditions: solution volume 0.35 mL, AC voltage 40 Vrms, AC time 10 s, DC voltage 350 V, pulse width 30 µsec, pulse interval 0.5 s, pulse number 3, decay rate 10%, no polarity switching, postfusion 7 s, and decay sine wave ON. Following fusion, cells were suspended in serum-containing RPMI1640 medium and incubated for 10 min to allow cell membrane recovery. The cells were then resuspended in RPMI1640 medium supplemented with 10% fetal bovine serum and HAT (hypoxanthine, aminopterin, and thymidine) and seeded into a 96-well plate. After approximately 10 days of culture, hybridomas were selectively grown, and the supernatant was collected for screening as described below.

Hybridomas producing the desired antibodies were screened by ELISA using peptides RRRGR [pS] PRRRTP [pS] PRRRRSQ [pS] PRRRRSQSRESQC and CRRRGRSPRRRTPSPRRRRSQ [pS] PRRRRSQSRESQ, along with the non-phosphorylated peptide RRRGRSPRRRTPSPRRRRSQSPRRRRSQSRESQC. Fifty μL of diluted antigen was added to each well of a Nunc multimodule plate and incubated overnight at 4–8°C. After removing the antigen solution, 100 μL of blocking solution (0.1% casein, 1 mM EDTA, PBS) was added to each well and incubated at room temperature for 1 h to immobilize the antigen.

Next, 100 μL of culture supernatant diluted in reaction solution (0.1% casein sodium, 1 mM EDTA, PBS) was added per well and incubated at room temperature for 1 h. Wells were washed with PBS containing 0.05% Tween-20, followed by the addition of 50 μL of horseradish peroxidase (HRP)-labeled anti-mouse IgG Fc-specific antibody, which was incubated at room temperature for 1 h. After washing again with PBS containing 0.05% Tween 20, 50 μL of TMB solution was added to develop color. The reaction was stopped with 1 M H_2_SO_4_, and absorbance was measured at 450 nm. Hybridomas reacting with the non-phosphorylated peptide RRRGRSPRRRTPSPRRRRSQSPRRRRSQSRESQC were excluded, ensuring antibody-producing hybridomas exhibited the desired specificity. Selected hybridomas underwent single cloning by limiting dilution to establish stable antibody-producing hybridomas.

The established hybridomas were cultured in a serum-free medium (Hybridoma-SFM, Gibco, Tokyo, Japan) for acclimation. Once acclimated, cells were expanded to 50 mL in a T75 flask. When cell density reached approximately 5 × 10⁵ cells/mL, they were transferred to a culture bag (Nipro, Osaka, Japan) containing 500 mL of serum-free medium. After 2–4 weeks of culture, the supernatant was collected, and the medium was applied to a column packed with Protein G Sepharose (Cytiva, Tokyo, Japan). Bound antibodies were eluted with a pH 3 buffer and neutralized immediately with 2 M Tris (pH 8). The buffer was then exchanged with PBS using a desalting column.

*iTACT HBcrAg, HBcAg, pHBcAg CLEIA*

All reactions from pretreatment to measurement were conducted using the fully automated chemiluminescent enzyme immunoassay system, LUMIPULSE L2400. HBcrAgs included translation products HBeAg and p22cr (PreC), derived from precore mRNA (pc-mRNA), as well as HBcAg, which included post-translationally modified forms from pgRNA transcribed from cccDNA. During the pretreatment process of the iTACT-HBcrAg CLEIA, common epitopes of HBcrAgs were exposed by denaturation and subsequently captured by mAbs HB44, HB124, HB114, and HB61, which were bound to magnetic particles during the primary reaction. For detection in the HBcrAg measurement system, mAbs HB91 and HB110, which recognize the common sequences across all HBcrAg molecules, were utilized. HBcrAg, HBcAg, and pHBcAg were ALP-labeled using Fab fragments derived from HB91, HB110, HB50, and B1126, respectively. The calibrators employed were recombinant HBeAg expressed in *E. coli* for HBcrAg, recombinant HBc antigen expressed in *E. coli* for HBcAg, and recombinant ProHBeAg phosphorylated with recombinant SRPK1 for pHBcAg (3, 4). Recombinant His-tagged SRPK1 was prepared through extraction from *E. coli* cells expressing the protein, followed by purification using nickel column affinity chromatography and gel filtration. The purified SRPK1 was reacted with ProHBeAg as previously described, with the reaction mixture being further purified in the presence of a dephosphorylation inhibitor and EDTA and stored at −80°C. The diluted calibrators were subsequently measured using iTACT, and standardization was performed based on HBcrAg measurement. The standardized values were then used to calculate quantitative results.

*OptiPrep density gradient ultracentrifugation*

OptiPrep (iodixanol: 60% (w/v) in water, Serumwerk Bernburg, Bernburg, Germany) was mixed with 6-fold concentrated phosphate buffered saline without calcium and magnesium (PBS (-), Fujifilm-Wako Pure Chemical, Osaka, Japan) to prepare 50% OptiPrep/PBS (-). This solution was further diluted with PBS (-) to prepare 10%, 20%, 30%, and 40% OptiPrep/PBS (-) solutions. Gradients were formed by layering equal volumes of these diluted solutions in ultracentrifuge 13PA tubes (Eppendorf, Tokyo, Japan). After a 30-min equilibration at room temperature, 100 or 300 μL of sample was applied to the top. Ultracentrifugation was conducted at 33,400 rpm, 4°C, for 16 h using a Himac CP100NX (Eppendorf HIMAC Technologies, Hitachi-Naka, Japan).

*WB analysis of OptiPrep density gradient ultracentrifugation* ***fractions***

Aliquots of the fractions were diluted in SDS sample buffer containing a phosphatase inhibitor cocktail (Nacalai Tesque, Kyoto, Japan) and neutralized TCEP (Tokyo Chemical Industry, Tokyo, Japan). Samples were heat-denatured at 98℃ for 5 min and then loaded along with a pre-stained marker (Bio-Rad, Tokyo, Japan) onto a 15% polyacrylamide gel (16 cm length; D.R.C., Tokyo, Japan). Electrophoresis was performed at a constant voltage of 220 V for 3 h. The gels were sectioned at the 37.5 kDa position of the pre-stained marker, and the lower gel portions were wet-transferred onto nitrocellulose membranes using 20% methanol-Tris-Glycine buffer. Membranes were blocked with Bullet Blocking One mixture (Nacalai Tesque) and incubated with biotinylated Fab fragments from mAbs at a concentration of 1 µg/mL, diluted in a 20-fold diluted Blocking One mixture. Following a 1-h incubation, the membranes were washed three times with TBS-T (0.1% Tween 20 in Tris-buffered saline) and probed with streptavidin-HRP diluted 1:100,000 in a 20-fold diluted Blocking One mixture. After five additional washes with TBS-T, the membranes were developed using SuperSignal West Femto substrate (Thermo Scientific, Tokyo, Japan). Chemiluminescent signals were detected using a chemiluminescence image analyzer, Fusion (Vilber Lourmat, Collégien France).

*Determination of PC and BCP mutations*

The direct-sequencing procedure was applied to determine mutations in PC and BCP using the amplified fragment carrying the core promoter and precore or core regions by PCR with hemi-nested primers described previously (5).

**Supplementary figures**

Number of supplementary figures: 5

Figure S1 p8

Figure S2 p10

Figure S3 p11

Figure S4 p12

Figure S5 p13


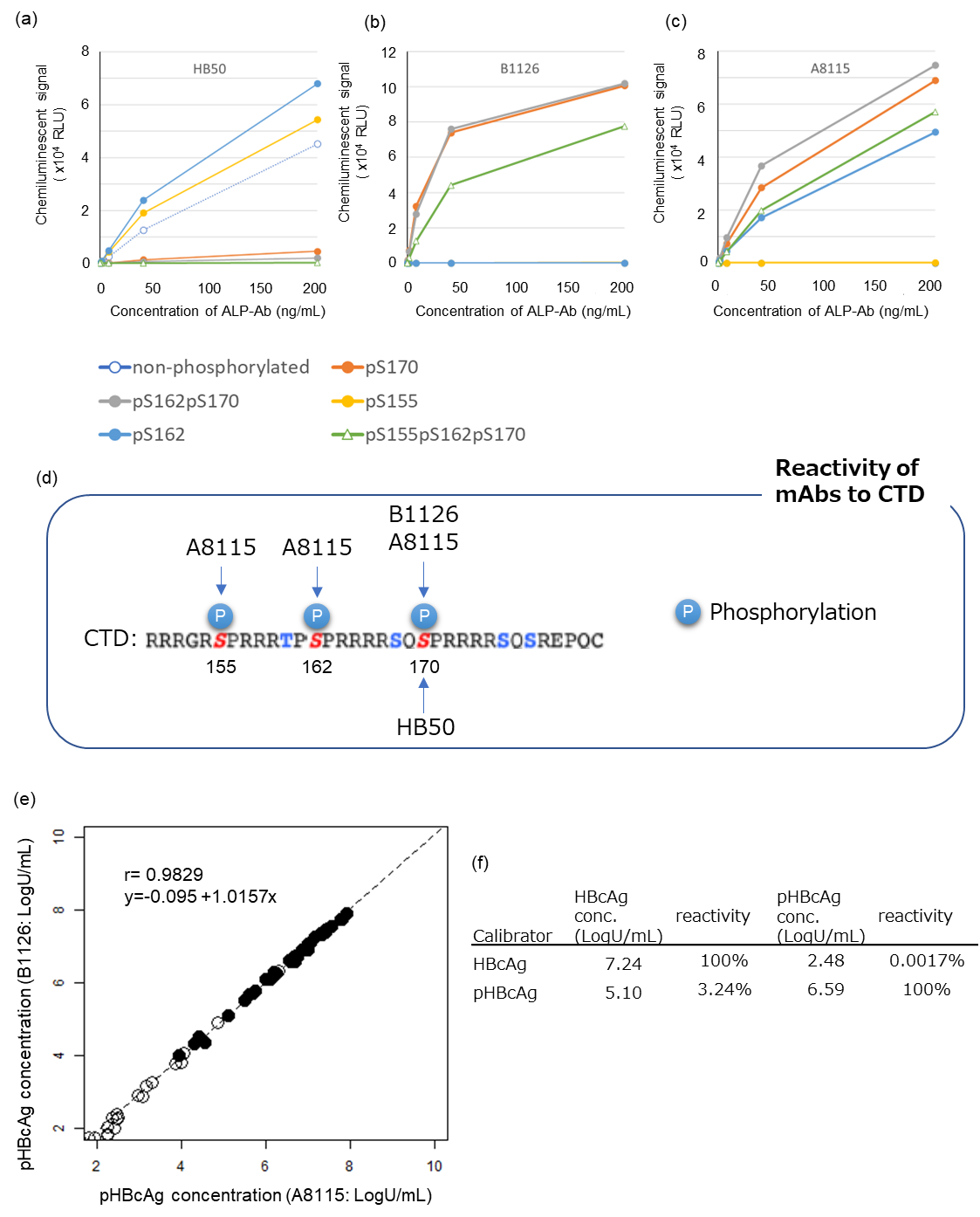

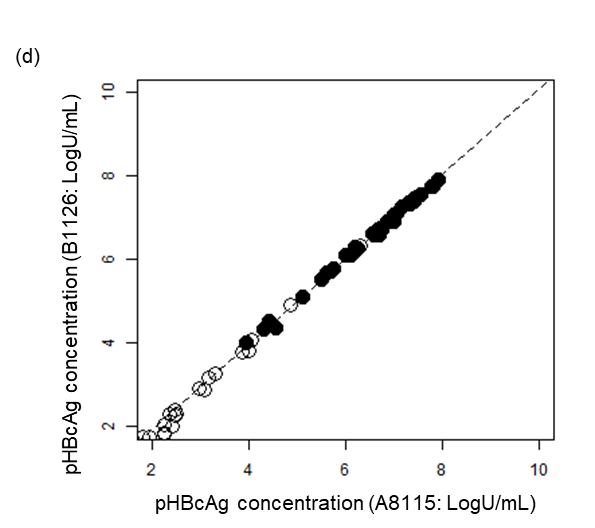

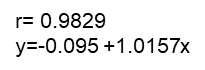


**Figure S1**. Reactivity of monoclonal antibodies to the C-terminal domain (CTD) of hepatitis B core antigen (HBcAg). Reactivities of alkaline phosphatase-conjugated Fab fragments (ALP-Ab) from monoclonal antibodies (mAbs) HB50 (a), B1126 (b), and A8115 (c) to various CTD peptides with phosphorylated serine residues at specific positions, as well as to the non-phosphorylated CTD peptide. The corresponding phosphorylated positions are noted in the legends provided at the bottom of the panels. Panel (d) shows the correlation between two pHBcAg assays using mAbs A8115 and B1126. The concentration of pHBcAg in HBeAg-positive samples (black circles) and HBeAg-negative samples (open circles) is plotted with A8115 on the horizontal axis and B1126 on the vertical axis. The Pearson correlation coefficient and the Passing-Bablok regression equations are displayed in the inset.


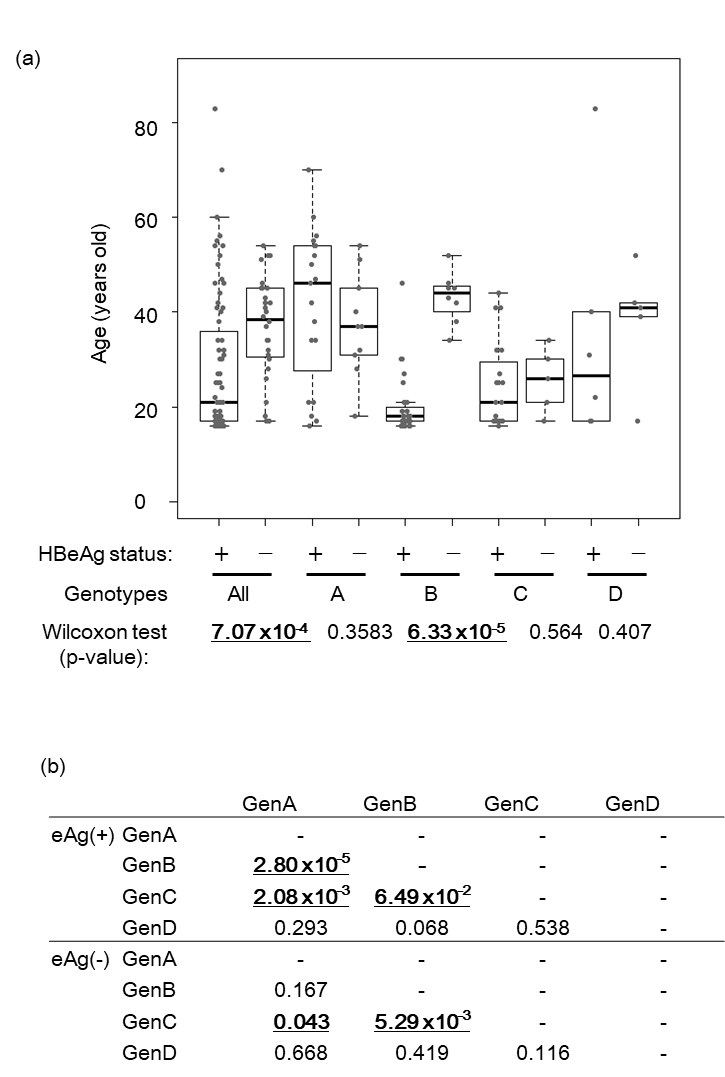
**Figure S2.** Age bias in purchased plasma specimens among genotypes. (a) Age bias between eAg(+) and eAg(–) cases within the purchased plasma specimens. Ages in eAg(+) and eAg(–) cases are plotted as box plots. Statistical values for each group are provided below the panel. The *p*-values from the Wilcoxon rank-sum test comparing ages between eAg(+) and eAg(–) groups are displayed, with values below 0.05 highlighted in bold and underlined. (b) Wilcoxon rank-sum test for comparison of median age of each genotype in eAg(+) and eAg(–) cases. *p*-values <0.05 are indicated in underlined bold text.


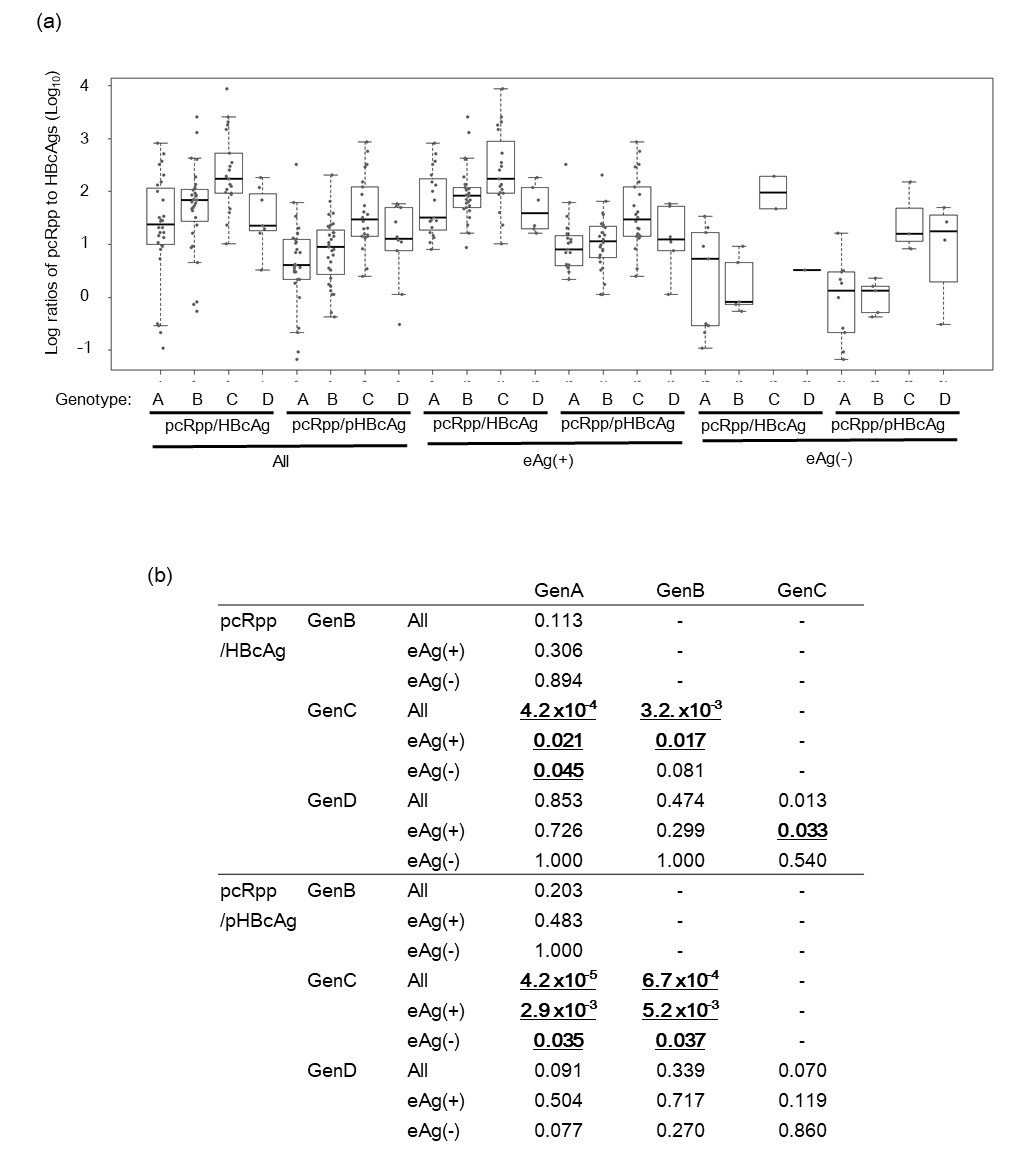
**Figure S3.** Distribution of ratios among hepatitis B core-related antigen (HBcrAg), hepatitis B core antigen and phosphorylated HBcAg (pHBcAg). Panel (a) compares the distribution of the ratios of pcRpp levels to HBcAg levels across HBV genotypes. The data for all, eAg(+), and eAg(–) cases are shown as box plots on a log_10_ scale in the upper panel. Panel (b) presents the results of the Wilcoxon rank-sum test for comparisons of the ratios of pcRpp levels to HBcAg levels between genotypes. *p* <0.05 are highlighted in bold and underlined to indicate statistically significant differences.


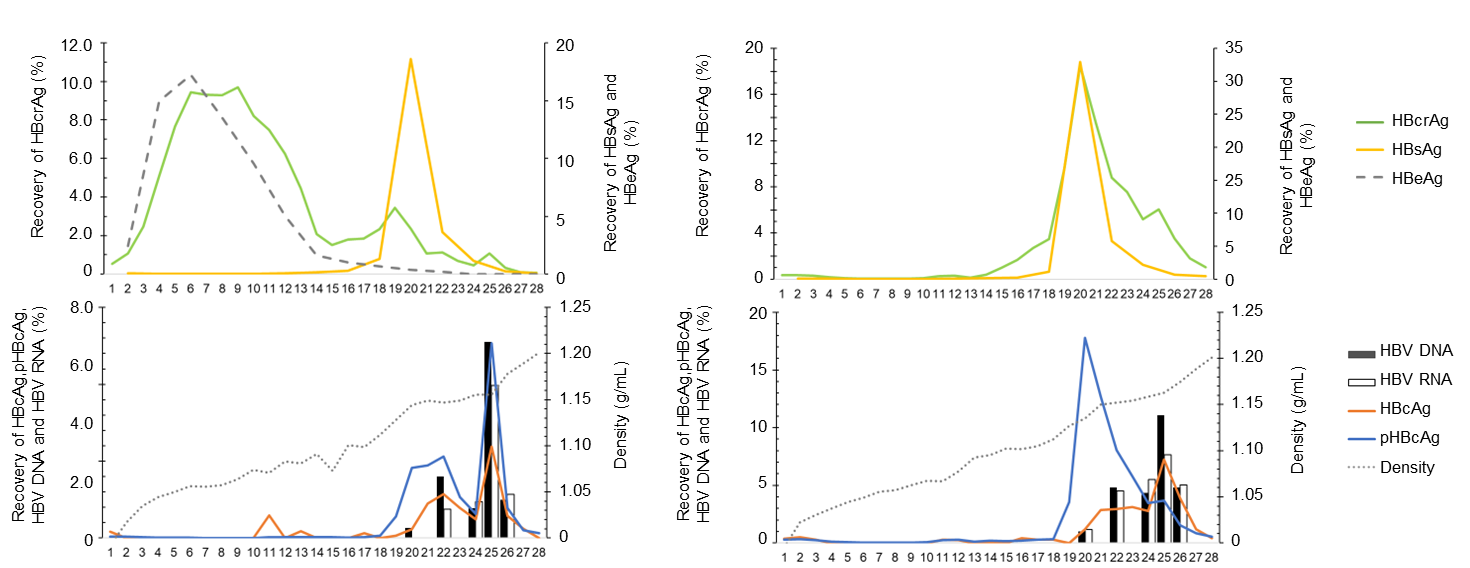


(a)

(b)

**Figure S4**. Fractionation of hepatitis B virus (HBV) biomarkers in hepatitis B e antigen (HBeAg) positive [eAg(+)], and hepatitis B antigen negative [eAg(–)], plasma specimens using Opti-Prep density gradient (ODG) ultracentrifugation. A genotype B eAg(+) plasma, HB220 (a), and a genotype B eAg(–) plasma, HB249 (b), were analyzed. Top panels illustrate the fractionation of HBV biomarkers. Recovery rate for hepatitis B core-related antigen (HBcrAg) is indicated on the left axis, and those for hepatitis surface antigen (HBsAg) and hepatitis e antigen (HBeAg) are on the right axis. Bottom panels display recovery rates of hepatitis core antigen (HBcAg), phosphorylated HBcAg (pHBcAg) and HBV DNA, HBV RNA. Their recovery rates in percentage are indicated on the left axis. The density of fractions is indicated on the right axis. The horizontal axes of all panels indicate the fraction numbers of ODG ultracentrifugation analysis. The marks of panels are shown at the right side of panels.


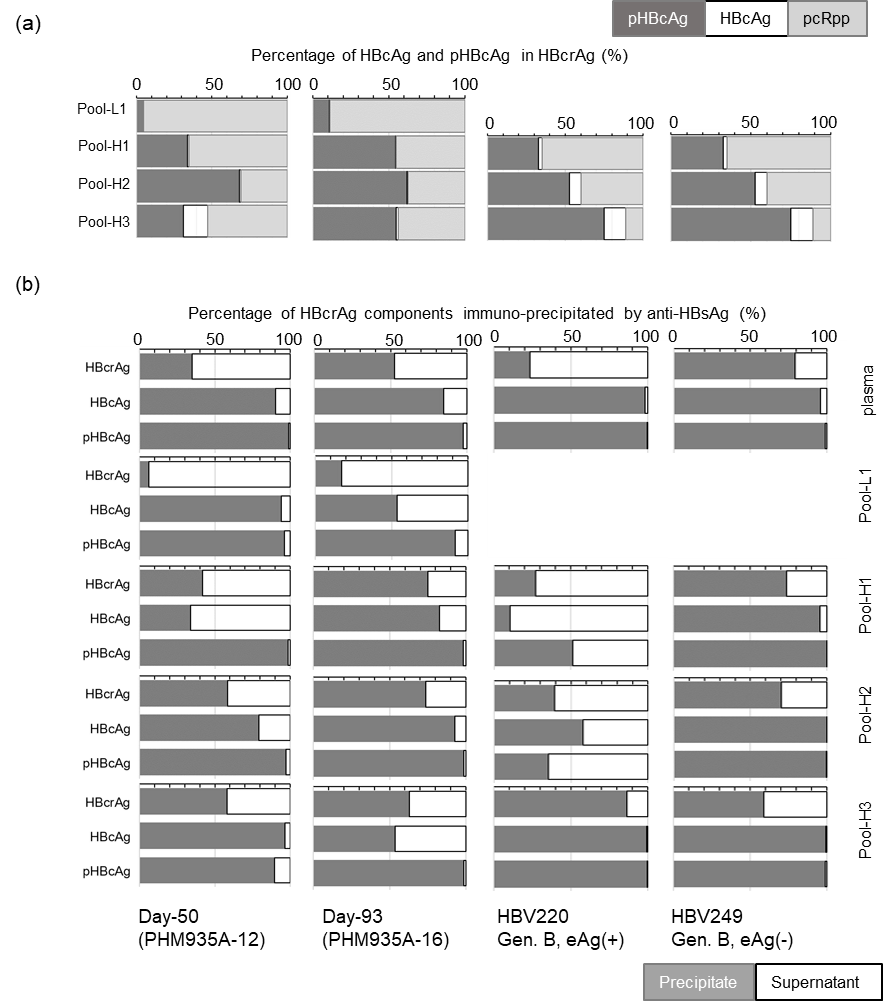


**Figure S5.** Analysis of enveloped HBcrAgs **components** fractionated by OptiPrep density gradient ultracentrifugation. (a) Percentage of HBcrAg components (HBcAg, pHBcAg, and pcRpp) in the PHM935A day-68 sample are represented as white, dark gray, and light gray bars, respectively. (b) The percentage of immunoprecipitated HBcrAg components in the same sample is shown. This includes anti-HBsAg immunoprecipitated HBcrAg, HBcAg, and pHBcAg, represented by gray bars.

**Supplementary tables**

Number of supplementary tables: 6

Table S1 p15

Table S2 p16

Table S3 p17

Table S4 p18

Table S5 p19

Table S6 p20

**Table S1.** Summary of the purchased 100 HBsAg-positive plasma specimens.

|  |  | eAg(+) | % | eAg(–) | % | Sum |
| --- | --- | --- | --- | --- | --- | --- |
| All |  | 72 | 72.0% | 28 | 28.0% | 100 |
| Sex | Male | 40 | 69.0% | 18 | 31.0% | 58 |
|  | Female | 32 | 76.2% | 10 | 23.8% | 42 |
| Genotype | A | 19 | 65.5% | 10 | 34.5% | 29 |
|  | B | 27 | 77.1% | 8 | 22.9% | 35 |
|  | C | 19 | 79.2% | 5 | 20.8% | 24 |
|  | D | 6 | 54.5% | 5 | 45.5% | 11 |
|  | F | 1 | 100% | 0 | 0% | 1 |

**Table S2.** Pearson correlation between each quantitative hepatitis B virus (HBV) biomarker.

|  |  |  | HBsAg | HBcrAg | HBcAg | pHBcAg | tHBcAg |
| --- | --- | --- | --- | --- | --- | --- | --- |
| All | HBV DNA | r | **0.219** | **0.874** | **0.900** | **0.895** | **0.901** |
|  |  | p-value | 2.88 ×10^–2^ | < 2.20 ×10^–16^ | < 2.20 ×10^–16^ | < 2.20 ×10^–16^ | < 2.20 ×10^–16^ |
|  | HBsAg | r | 1 | 0.102 | 0.176 | 0.164 | 0.172 |
|  |  | p-value | NA | 3.14 ×10^–1^ | 8.95 ×10^–2^ | 1.05 ×10^–1^ | 8.89 ×10^–2^ |
|  | HBcrAg | r |  | 1 | **0.896** | **0.933** | **0.931** |
|  |  | p-value |  | NA | < 2.20 ×10^–16^ | < 2.20 ×10^–16^ | < 2.20 ×10^–16^ |
|  | HBcAg | r |  |  | 1 | **0.970** | **0.980** |
|  |  | p-value |  |  | NA | < 2.20 ×10^–16^ | < 2.20 ×10^–16^ |
|  | pHBcAg | r |  |  |  | 1 | **0.999** |
|  |  | p-value |  |  |  | NA | < 2.20 ×10^–16^ |
| eAg(+) | HBV DNA | r | **0.365** | **0.750** | **0.790** | **0.762** | **0.770** |
|  |  | p-value | 2.19 ×10^-3^ | 1.83 ×10^-13^ | 1.33 ×10^-15^ | 4.42 ×10^-14^ | 1.64 ×10^-14^ |
|  | HBsAg | r | 1.000 | 0.219 | **0.361** | **0.339** | **0.347** |
|  |  | p-value | NA | 7.29 ×10^-2^ | 2.51 ×10^-3^ | 4.65 ×10^-3^ | 3.75 ×10^-3^ |
|  | HBcrAg | r |  | 1.000 | **0.814** | **0.850** | **0.849** |
|  |  | p-value |  | NA | < 2.20 ×10^–16^ | < 2.20 ×10^–16^ | < 2.20 ×10^–16^ |
|  | HBcAg | r |  |  | 1.000 | **0.952** | **0.965** |
|  |  | p-value |  |  | NA | < 2.20 ×10^–16^ | < 2.20 ×10^–16^ |
|  | pHBcAg | r |  |  |  | 1.000 | **0.999** |
|  |  | p-value |  |  |  | NA | < 2.20 ×10^–16^ |
| eAg(–) | HBV DNA | r | **-0.392** | **0.522** | **0.806** | **0.751** | **0.786** |
|  |  | p-value | 2.64 ×10^-2^ | 2.18 ×10^-3^ | 6.56 ×10^-7^ | 1.11 ×10^-6^ | 1.60 ×10^-7^ |
|  | HBsAg | r | 1.000 | **-0.451** | **-0.610** | **-0.480** | **-0.483** |
|  |  | p-value | NA | 9.59 ×10^-3^ | 9.47 ×10^-4^ | 6.34 ×10^-3^ | 5.97 ×10^-3^ |
|  | HBcrAg | r |  | 1.000 | **0.700** | **0.805** | **0.793** |
|  |  | p-value |  | NA | 6.91 ×10^-5^ | 4.81 ×10^-8^ | 1.06 ×10^-7^ |
|  | HBcAg | r |  |  | 1.000 | **0.893** | **0.935** |
|  |  | p-value |  |  | NA | 8.17 ×10^-10^ | 2.57 ×10^-12^ |
|  | pHBcAg | r |  |  |  | 1.000 | **0.993** |
|  |  | p-value |  |  |  | NA | < 2.20 ×10^–16^ |

Correlation coefficient in bold-typed letters indicate those with p-values below 0.05.

**Table S3**. Quantitative biomarker levels of the HBeAg seroconversion panel (PHM935A, B).

|  | Vendor data sheet | | | | |
| --- | --- | --- | --- | --- | --- |
| Member | Since 1st Bleed (days) | HBeAg C.O.I. | HBeAb C.O.I. | HBsAb C.O.I. | Roche PCR  log(cp/ml) |
| PHM935A-01 | 0 | 0.2 | NT | NT | <2 |
| PHM935A-02 | 2 | 0.2 | NT | NT | <2 |
| PHM935A-03 | 7 | 0.2 | NT | NT | <2 |
| PHM935A-04 | 9 | 0.2 | NT | NT | 2.78 |
| PHM935A-05 | 14 | 0.3 | NT | NT | 2.90 |
| PHM935A-06 | 16 | 0.2 | NT | NT | 2.70 |
| PHM935A-07 | 21 | 0.2 | NT | NT | 3.95 |
| PHM935A-09 | 28 | 0.3 | NT | NT | 4.90 |
| PHM935A-10 | 30 | 0.3 | NT | NT | 5.00 |
| PHM935A-11 | 35 | 1.4 | NT | NT | 5.60 |
| PHM935A-12 | 50 | 15.4 | NT | NT | 7.30 |
| PHM935A-13 | 66 | 17.8 | NT | NT | 6.70 |
| PHM935A-14 | 68 | 17.2 | NT | NT | >7.60 |
| PHM935A-15 | 85 | 14.9 | NT | NT | >7.60 |
| PHM935A-16 | 93 | 16 | NT | NT | 7.00 |
| PHM935A-17 | 100 | 10.8 | NT | NT | 6.30 |
| PHM935A-19 | 114 | 7.2 | NT | NT | 4.48 |
| PHM935A-20 | 121 | 6.5 | NT | NT | 4.30 |
| PHM935B-21 | 128 | 4.5 | 0.4 | 0.4 | 3.60 |
| PHM935B-22 | 135 | 4.3 | 0.5 | 0.4 | 3.00 |
| PHM935B-23 | 151 | 3.2 | 0.6 | 0.4 | 2.70 |
| PHM935B-24 | 165 | 2.4 | 0.5 | 0.5 | 2.90 |
| PHM935B-25 | 175 | 2.1 | 0.6 | 0.4 | 3.30 |
| PHM935B-26 | 189 | 1.7 | 0.6 | 0.5 | 2.90 |
| PHM935B-27 | 203 | 0.8 | 0.8 | 0.6 | <2 |
| PHM935B-28 | 217 | 0.6 | 0.9 | 0.7 | <2 |
| PHM935B-29 | 231 | 0.4 | 0.9 | 1.1 | <2 |
| PHM935B-30 | 246 | 0.3 | 1 | 1.6 | <2 |
| PHM935B-31 | 262 | 0.3 | 1.3 | 5.7 | <2 |
| PHM935B-32 | 273 | 0.3 | 1.2 | 6.7 | <2 |

NT: not tested

Underlined values: values above cut-off ones

**Table S4.** Cases under NA treatment with PC/BCP mutation analysis data.

| Mutation status | | HBeAg status | |  |
| --- | --- | --- | --- | --- |
| PC | BCP | eAg (+) | eAg(–) | Total |
| WT | WT | 5 | 1 | 6 |
| WT | MT | 3 | 4 | 7 |
| Mix | MT | 1 | 1 | 2 |
| MT | MT | 2 | 9 | 11 |
|  | Total | 11 | 15 | 26 |

**Table S5.** Summary of the HBsAg-positive plasma specimens analyzed by OptiPrep density gradient analysis.

|  | HBV RNA | HBV DNA | HBeAg status | Genotype | HBsAg | HBcrAg | HBcAg | pHBcAg |
| --- | --- | --- | --- | --- | --- | --- | --- | --- |
|  | Log_10_(cp/mL) | Log_10_(IU/mL) |  |  | IU/mL | LogU/mL | LogU/mL | LogU/mL |
| PHM935A day 50 | 4.58 | 7.50 | eAg(+) | A | 1,439 | 7.43 | 5.90 | 6.77 |
| PHM935A day 68 | 6.62 | 9.20 | eAg(+) | A | 114,515 | 8.92 | 7.68 | 8.49 |
| PHM935A day 93 | 4.17 | 7.04 | eAg(+) | A | 43,939 | 8.20 | 6.41 | 7.66 |
| HBV220 | 5.31 | 8.00 | eAg(+) | B | 15,085 | 8.10 | 6.31 | 7.10 |
| HBV249 | 5.07 | 7.68 | eAg(–) | B | 5,876 | 6.99 | 5.84 | 6.78 |

**Table S6** Summary of pooled fractions for anti-HBsAg immunoprecipitation

|  |  |  | Density of fraction-pool (g/mL) | |
| --- | --- | --- | --- | --- |
| Samples | Pooled fraction name | pooled fraction numbers | median | Average |
| PHM935A-12 | Pool-L1 | 10–12 | 1.075 | 1.074 |
| day 50 | Pool-H1 | 19–21 | 1.137 | 1.135 |
|  | Pool-H2 | 22–24 | 1.155 | 1.155 |
|  | Pool-H3 | 25–27 | 1.173 | 1.176 |
| PHM935A-14 | Pool-L1 | 11–13 | 1.080 | 1.079 |
| day 68 | Pool-H1 | 19–21 | 1.142 | 1.133 |
|  | Pool-H2 | 22, 23 | 1.152 | 1.152 |
|  | Pool-H3 | 24–26 | 1.158 | 1.164 |
| PHM935A-16 | Pool-L1 | 11–13 | 1.083 | 1.084 |
| day 93 | Pool-H1 | 19–21 | 1.141 | 1.139 |
|  | Pool-H2 | 22, 23 | 1.151 | 1.151 |
|  | Pool-H3 | 24–26 | 1.167 | 1.171 |
| HBV220 | Pool-H1 | 20, 21 | 1.147 | 1.147 |
|  | Pool-H2 | 22, 23 | 1.148 | 1.148 |
|  | Pool-H3 | 24–26 | 1.155 | 1.163 |
| HBV249 | Pool-H1 | 19–21 | 1.135 | 1.137 |
|  | Pool-H2 | 22–24 | 1.154 | 1.155 |
|  | Pool-H3 | 25–27 | 1.175 | 1.175 |

**Supplementary References**

1. Usuda S, Okamoto H, Iwanari H, Baba K, Tsuda F, Miyakawa Y, Mayumi M. 1999. Serological detection of hepatitis B virus genotypes by ELISA with monoclonal antibodies to type-specific epitopes in the preS2-region product. J Virol Methods 80:97–112.

2. Tadokoro K, Kobayashi M, Yamaguchi T, Suzuki F, Miyauchi S, Egashira T, Kumada H. 2006. Classification of hepatitis B virus genotypes by the PCR-Invader method with genotype-specific probes. J Virol Methods 138:30–39.

3. Daub H, Blencke S, Habenberger P, Kurtenbach A, Dennenmoser J, Wissing J, Ullrich A, Cotten M. 2002. Identification of SRPK1 and SRPK2 as the Major Cellular Protein Kinases Phosphorylating Hepatitis B Virus Core Protein. J Virol 76:8124–8137.

4. Heger-Stevic J, Zimmermann P, Lecoq L, Böttcher B, Nassal M. 2018. Hepatitis B virus core protein phosphorylation: Identification of the SRPK1 target sites and impact of their occupancy on RNA binding and capsid structure. PLoS Pathog 14:e1007488.

5. Sugauchi F, Mizokami M, Orito E, Ohno T, Kato H, Suzuki S, Kimura Y, Ueda R, Butterworth LA, Cooksley WGE. 2001. A novel variant genotype C of hepatitis B virus identified in isolates from Australian Aborigines: complete genome sequence and phylogenetic relatedness. J Gen Virol 82:883–892.
